# The relationship between education levels, lifestyle, and religion regarding the prevalence of myopia in Israel

**DOI:** 10.1186/s12886-021-01891-w

**Published:** 2021-03-16

**Authors:** Sharon Armarnik, Maya Lavid, Sharon Blum, Tamara Wygnanski-Jaffe, David B. Granet, Michael Kinori

**Affiliations:** 1 The Goldschleger Eye Institute, Sheba Medical Center, Sackler Faculty of Medicine, Tel Aviv University, 521 Tel Hashomer, Israel; 2 Ratner Children’s Eye Center at the Shiley Eye Institute, University of California, San Diego, 9415 Campus Point Drive, La Jolla, San Diego, CA 92093 USA

**Keywords:** Myopia, Ultra-orthodox, Accommodative effort, Education, Near work

## Abstract

**Background:**

The ultra-Orthodox Jewish community has a unique lifestyle including minimal outdoor activity and intense, prolonged nearby work, beginning at a very young age. Their prevalence of myopia is extremely high. This paper provides a unique insight into the attitudes of this community towards myopia.

**Methods:**

Ultra-Orthodox Jewish parents of children who came to the pediatric ophthalmology clinic in one tertiary care and two community centers in ultra-Orthodox-oriented cities were given a questionnaire. Demographic information, along with myopia prevalence in the family, was gathered. In addition, their attitudes and common knowledge regarding myopia were investigated.

**Results:**

161 questioners were collected, mostly completed by mothers (*n* = 110, 68%). The average number of children per family was 6 (range 1–16). In 148 families (92%) at least one of the parents has myopia. The average parent refraction was − 4.5 diopters (range − 0.5 to 15 diopters). Out of 935 children, 410 (44%) wore glasses. Twelve parents (7%) believe that myopia is a disease and 94 (58%) reported that they are concerned because their child wears glasses. Twenty-four (15%) believe that glasses are a sign of a high education level. Regarding treating myopia progression, 144 (89%) think that myopia progression should be treated, but only 36 (22%) are aware of the available treatments for it.

**Conclusion:**

This study examines an insular community with a very high incidence of myopia. In this community most parents think that myopia progression should be treated but most of them are unaware of the currently available treatments.

## Background

It is estimated that the global prevalence of myopia (≤ − 0.50 D) and high myopia (≥ − 5.00 D) will increase to a predicted number of nearly 5 billion people (almost 50% of the world population) and 1 billion people, respectively, by 2050 [1]. In some regions, myopia prevalence is already extremely high, reaching 69% at 15 years of age in east Asia (and up to 86% among Singaporean-Chinese) with higher prevalence in children from urban environments compared with those from rural environments [2]. Many predisposing factors to myopia development and progression have been studied. It is known that both genetics and environmental factors play a role in its development. On the one hand, some reports have shown that the role of genetics is more substantial in myopia development [3, 4], as indicated by the discovery of a myopia susceptibility gene found by wide genome sequencing in the Ashkenazi Jewish population [5, 6]. These mutations however have not yet been linked to myopia in Ultra-Orthodox communities. On the other hand, the environmental elements in myopia development and progression can be addressed in an attempt to halt its progression. Low levels of outdoor activity and increased nearby work are well established risk factors for myopia; however, additional environmental risk factors have been reported [7]. A prolonged near task has been shown to cause axial elongation [8], and it has been speculated that near work can differently influence its progression in each refractive group [8, 9].

The public Jewish educational system in Israel can generally be divided into secular and Orthodox school systems, which differ in many fundamental aspects, especially the subjects taught and emphasized. The Orthodox community can be subdivided into Orthodox and ultra-Orthodox communities. Ultra-Orthodox Jews in Israel have a unique educational system that involves intensive sustained near-work activity and a prolonged accommodative effort to read small letters beginning at a younger age, compared with the other educational systems. Children in the ultra-Orthodox streamsection do not enroll in kindergarten but instead commence their formal schooling with an emphasis on intense studying and reading, starting at the age of three years. Boys and girls study in a separate school system and they have different curricular activities. The number of study hours, mainly for boys, gradually increased to up to 16 h a day for teenagers. Their sustained near work is characterized by increased accommodative effort, accompanied by rocking movements back and forth to increase their concentration. There is emphasis on reading texts with varying font sizes, which may be as small as one millimetre in height [10]. Most of the curriculum is also characterized by intensive reading of small printed religious texts in class, and by few extracurricular activities or outdoor programs [11]. Adolescents in ultra-Orthodox schools are less exposed to technology, and specifically to devices with screens, at school and in their everyday life [9]. The vast majority of ultra-Orthodox teenagers either do not have cellular phones, and if they do, these phones are not smart phones and the internet connection is disabled.

Several publications on this topic have shown that this type of educational system is associated with increased myopia severity [11–13]. The prevalence of myopia in young Orthodox males was found to be around 72.5–81.3% [10, 12] On the other hand, in China, the incidence of myopia among students is among the highest of any cultural or ethnic group, and it affects both genders equality [14]. It may emphasis the importance of the overall balance of near work and time outdoors as well as the different curricular activities between the genders in the Ultra Orthodox community.

An ultra-Orthodox teenager, especially a male, who does not wear glasses, is considered extremely unique in this community.

In this study we aimed to assess the relationship between education and life style within the ultra-Orthodox community in Israel as well as their knowledge and attitude towards Myopia.

## Materials and methods

This study was approved by the local ethics committee of Sheba Medical Center, named Sheba Medical Center IRB committee, and the “Meuhedet” public health plan. Our local IRB committee approved this research with a waiver of informed consent since this research involves no more than minimal risk to subjects. All methods were performed in accordance with the relevant guidelines and regulations. Ultra-Orthodox Jewish parents of children who came to the pediatric Ophthalmology clinic in one tertiary care center and two community centers in Ultra-Orthodox oriented cities, namely, Bnei Brak and Qiriat Sefer, where one of the authors (MK) works, were given a questionnaire to complete. Parents who did not define themselves as ultra-Orthodox were excluded. Parents of children with hypermetropia were not given questionnaires. The questionnaire was written in Hebrew. Sometimes the participants needed help to explain the language in the questionnaire, since the mother tongue of some ultra-Orthodox parents is Yiddish (a language used by Jews in central and eastern Europe before the Holocaust). Demographic information included parent’s gender, age, glasses wear and medical ophthalmology history including refractive surgery or other eye surgery needed, data on myopia prevalence of the spouse and their children. The attitude towards myopia and common knowledge regarding myopia and myopia progression treatment options was collected. Statistical analysis was carried out using Microsoft Excel 2013 (Microsoft Corporation, Redmond, WA, USA).

## Results

### Demographics

A total of 161 questionnaires were collected, mostly completed by mothers (*n* = 110, 68%). Forty-one parents (25%) were under 31 years of age, 71 (44%) were between 31 and 40 years, 41 (26%) were between 41 and 50 years, and 3 (2%) were 50 years or above. There were more fathers wearing glasses than mothers (88% vs. 63%, *p* > 0.01). The average number of children per family was 6 (range 1–16) and 84 families (52%) had 6 children or more. The total number of children of parents who completed the questionnaire was 935.

### Myopia prevalence

In 148 families (92%) at least one parent has myopia and in 83 families (52%) both parents are myopic. The parental average refraction was − 4.5 diopters (range − 0.5 to -15D). Eight (5%) of the parents had previous refractive surgery. Out of 935 children, 410 (44%) wore glasses.

### Knowledge of and attitudes towards myopia

Twelve parents (7%) indicated that they believe that myopia is a disease. One hundred and twenty-four (77%) believe that it is a genetic disorder. Ninety-four (58%) reported that they are bothered by the fact that their child wears glasses and 2 (1%) believe that wearing glasses causes a social problem. Fifty-eight (36%) consider Myopia a financial burden for the parents. Twenty-four (15%) believe that glasses are a sign of a high education level.

### Prevention and treatment of myopia

Myopia progression was associated with prolonged reading by 49 (30%) and with exposure to screen use by 107 (66%). Fifty-nine (37%) think that wearing glasses causes the prescription to increase and 53 (33%) believe that a lowered prescription should be given to prevent myopia progression. Outside activity was associated with a decreased prevalence of myopia by 13 (8%). Regarding treating myopia progression, 144 (89%) think that myopia progression should be actively treated, but only 36 (22%) are aware of the currently available treatments for it. Eighty-three (52%) would be willing to apply eye drops for a long period of time and 65 (40%) indicated they would let their child wear contact lenses to stop myopia progression.

## Discussion

The overall prevalence of myopia in Israel has been reported to be 28.3% [15] and 26.2% [16] among participants aged 16–19 years. The data for both of those studies were taken from a computerized database of the Israeli Defense Forces, since all 16-year-old Israelis are considered candidates for military service and therefore are obliged by law to appear at the Israel Defense Forces induction center. However, as the authors of both studies noted, ultra-Orthodox teenagers are extremely under-represented in the Israeli Defense Forces, since most of them are currently exempt from military service by law.

According to the Israeli Central Bureau of Statistics (CBS), the ultra-Orthodox community in Israel represents 11% of the total population and 14% of the Jewish community in Israel [17]. However, there is a much higher birth rate among ultra-Orthodox families: the average number of child births for the average ultra-Orthodox woman is 6.91, compared to 2.14 for a non-Orthodox Jewish woman. Therefore, it is not surprising that 25.7% of children under the age of 14 years and who live in Israel belong to the ultra-Orthodox Jewish community. Since the incidence of myopia is extremely high in this population group, it is reasonable to assume that the prevalence of myopia in Israel is higher than mentioned previously and will continue to increase, since according to the Israeli CBS, the percentage of ultra-Orthodox Jews in Israel is expected to increase from 11% today to 32% by the year 2065. In our cohort, the average number of children per family was 6, but most of these parents, who are in their 20s or 30s, are still in their reproductive years. For example, in Kiriat Sefer, one of the sites from which questionnaires were collected, the average family has 7.6 children [17].

**Fig. 1:**
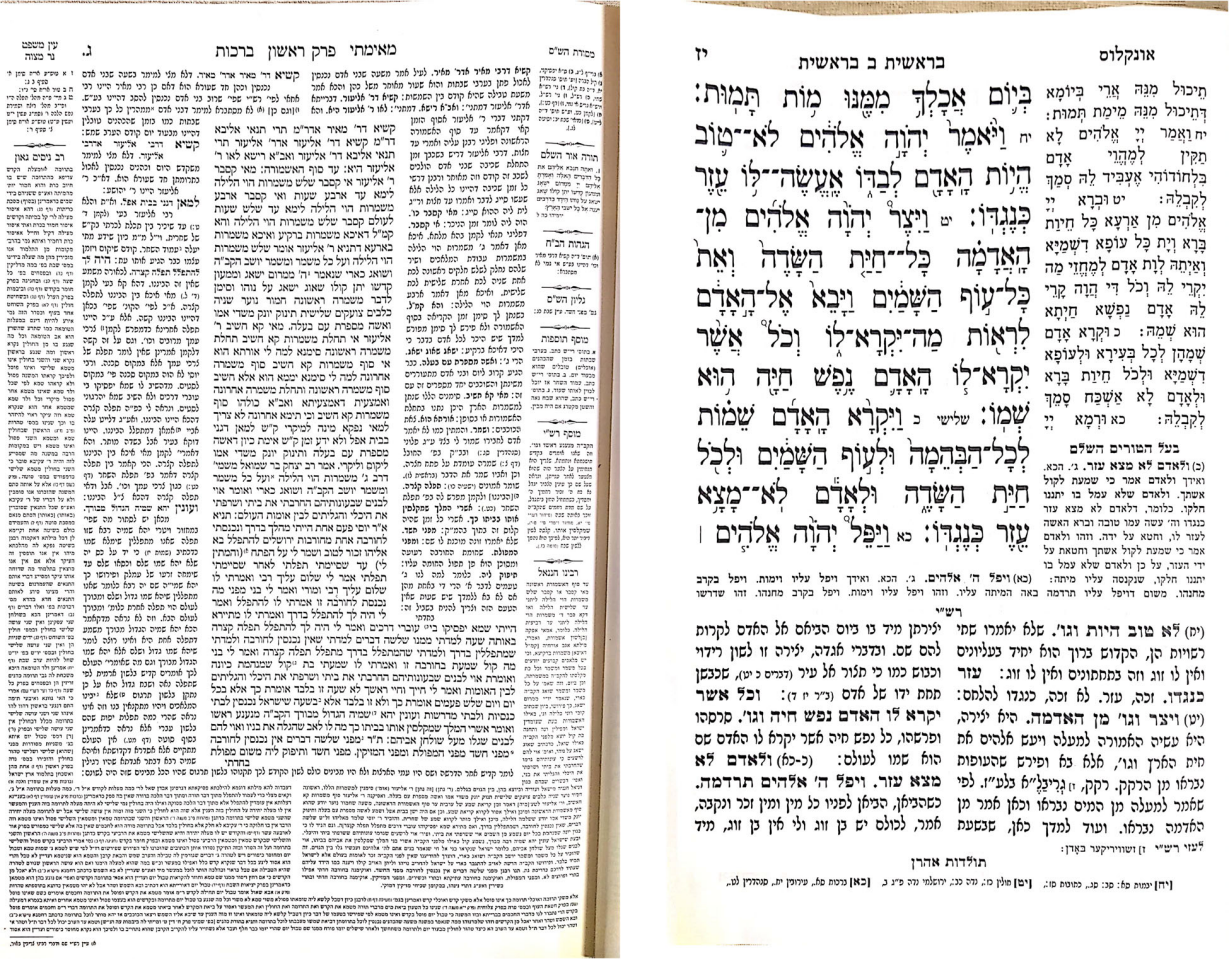
A page from a typical “Chumash” textbook for 8-year-old boys and girls (right) and from “Babylonian Talmud” for 13-year-old boys (left) in the Ultra-Orthodox community

The ultra-Orthodox run one of the three educational systems in Israel and differ in some ways from the western traditional education systems. Their system involves intensive sustained near-work activity and a prolonged accommodation effort beginning at 3 years of age [18]. As already mentioned, ultra-Orthodox schools focus mainly on intensive reading of religious texts and their learning involves swaying movements (also termed shockeling) back and forth in an effort to increase their concentration levels. This frequent changes in accommodation owing to the swaying habit during study. Most of the texts that the children read are printed in relatively small font, which may be as small as 1 mm in height (Fig. 1). Male and female students are separated in ultra-Orthodox schools, and they have different curricula and study conditions with the boys having longer study hours viewing smaller print. Zylbermann et al. showed that the prevalence of myopia in Orthodox teenager’s males was more than twice the percentage in females’ in Israel with a higher mean of the myopia [10]. In our cohort we also found higher percentage of myopia in fathers compared to mothers. The number of the boys’ study hours is gradually increased up to 16 h a day. In addition, adolescents in ultra-Orthodox schools are less exposed to technology, and specifically to devices with screens at school and in their everyday life. In addition, since this specific population is relatively isolated, with less access to the internet and public media, there is less awareness of the need for routine eye exams recommended for the general population. It is not uncommon for these children not to be examined by an ophthalmologist until the child or his teacher notices a vision issue. Even then, some parents go to a local optometrist, who prescribes glasses if needed, and their child is never examined by an ophthalmologist; therefore, they are less aware of the myopia control methods currently available. Moreover, since these children study in a room (“chaider”) with many hours devoted to reading and discussing religious texts (as opposed to a standard classroom with a black board that could be far away), these children may not complain of blurred vision at a distance at all, even if myopia is present. It is not uncommon to find significant myopia of minus 2–3 diopters and even more with the first exam, and with very few complaints from the child. In addition, in this community it is not acceptable to prescribe glasses in cases of low myopia such as minus 1 or even 2 diopters. One reason could be the minimal impact on their everyday life (“why does he/she need to see so far? He/she reads just fine”). This was noted, along with the belief that just wearing glasses would cause the myopia to increase, which was mentioned by 37% of the parents. This claim is debatable. Some studies showed that continuous correction of myopia results in a linear progression [19–21], while others showed that undercorrection of myopia will actually increases it [22, 23]. Therefore, the prevalence of myopia in this community could not be measured by the number of children wearing glasses or by a database, since the prevalence would be much higher. In addition, there is no available database for this group, since most of ultra-Orthodox children are not being examined if they are “asymptomatic”, and if they are, most of them will see a local optometrist and not an ophthalmologist, who are much less available (and who do not necessarily prescribe glasses in their practice).

Israel has a relatively well-developed public health system and the cost for children’s glasses is mostly covered (generally one pair of glasses per calendar year) by public health insurance, with a minimal amount of around 60 shekels (~$17) for a standard pair. However, in our cohort 36% felt that buying glasses for their children is a financial burden. This is probably because children tend to break or lose glasses more often than adults do, together with the fact that in some families more than 10 siblings wear glasses, which could become a financial burden, because these families have a mean income that is less than 70% of the national average [17].

It was interesting to find that a large proportion of ultra-Orthodox parents believe that myopia progression is associated with screen use rather than with reading. Since most children in this community are not exposed to smartphones or computer screens, we speculate that this may be explained by the aversion of this community to modern technology. Only 13% of parents in our cohort believe that myopia progression is associated with increased indoor time, even though spending time outside in natural light has been shown to decrease its progression [24, 25].

Lastly, almost 90% of ultra-Orthodox parents believe that myopia progression should be actively halted if possible, but only 22% are aware of its current treatment. However, more than half are willing to treat their children with daily eye drops in order to stop myopia progression and less than that number of parents let their child wear special contact lenses for this purpose. Since the cost of low-concentration atropine eye drops is covered by the Israeli public health system and special contact lenses such ortho-K or multifocal contact lenses are not and are expensive to purchase, treatment with multifocal contact lenses is generally not suitable, again, taking in consideration that the average ultra-Orthodox family will need to treat a number of children simultaneously. Other treatment options in the literature regarding halting myopia progression such as partial correction of hyperopia of a child at risk of developing myopia [26] were not addressed in this questionnaire.

This study has several limitations. First, not all parents were willing to complete the questionnaire, mainly due to privacy issues. Second, a minority of children who wear glasses are not myopic but are hyperopic. However, in this community the parents are generally well aware of the difference between hyperopic and myopic correction: hyperopic glasses are called “Magdelet” (=makes an object larger) as opposed to “regular glasses” or “Maktenet” (=makes an object smaller). Parents of a child with hyperopia were not given a questionnaire and if it was filled out, it was later excluded. In addition, this study’s main aim was not to measure the prevalence of myopia in this community but rather, to assess their attitude towards it. Lastly, since the study population consisted only of parents who came with their children to an ophthalmologist, a selection bias is likely.

## Conclusions

This study served as a rare opportunity to examine an insular community with a very high incidence of myopia. In this tight-knit community, most parents think that myopia progression should be treated if possible, yet most of them are unaware of the currently available treatments. However, most ultra-Orthodox parents think that this health issue should be addressed and are willing to treat their children with a daily eye drop regimen for an extended period of time. Israel may need to increase education levels in the Ultra-Orthodox community in the field Myopia, including modern understandings of what causes it, and how to control it. Further study on this population may help to advance our understanding of the mechanisms underlying myopia and hence, find effective ways to treat it.

## Data Availability

The data set used and analysed during the current study is attached as an excel table.

## Abbreviation

CBS: Central Bureau of Statistics
